# The relationship between student employability and student engagement: working toward a more unified theory

**DOI:** 10.3389/fpsyg.2014.00238

**Published:** 2014-03-20

**Authors:** Carl Senior, Peter Reddy, Rowena Senior

**Affiliations:** ^1^School of Life and Health Sciences, Aston UniversityBirmingham, UK; ^2^Centre for Learning and Innovation in Professional Practice, Aston UniversityBirmingham, UK

**Keywords:** engagement, employability, students, university, retention

As the field of Psychology evolves there is much interest in understanding how educational practices can be shaped to facilitate the recruitment and engagement of students (Halpern and Hakel, 2010). Highlighting the multiplicity of research approaches within Psychology is one way in which to advance the field. Indeed, in her recent article Rees (2013) argues that the multiplicity of research topics and methodologies is especially important to the discipline and that “promoting and highlighting this should be considered as a potentially effective recruitment strategy” (Rees, 2013 p. 1). Rees argues that the tension in Psychology between the traditional use of scientific method and the inappropriateness of this approach to many of the phenomena of interest create a dialectic that is both an opportunity and a threat. A threat in that such conflict may be off-putting but an opportunity in that methodological pluralism may be a selling point. Rees goes on to support Henriques (2013) in arguing that attempting to unify Psychology around a commitment to research methodology is flawed and that conceptual unification is necessary.

It is Rees's argument that highlighting the unique nature of empirical Psychology is a necessity for student recruitment that is the focus of this paper. Rees suggests that only by highlighting the unique plurality of approaches to our incoming students can we ensure that they become aware of the dialectic that is idiosyncratic to the field of Psychology. By further developing an understanding of this dialectic it is possible that our students will come equipped with a more realistic understanding of what are the unique contributions that Psychology can make to our everyday lives. It is this latter aspect of the Rees model that has great utility and the potential for significant impact on the recruitment, engagement, and subsequent retention of talented undergraduate students. However, such a model only describes one mechanism for student engagement and as such it needs to be developed further before it can contribute to the “unified theory which focuses on providing a conceptual map of the full breadth of psychological enquiry” (Rees, 2013 p. 1). In order to do this one must consider the unique mindset of the students themselves as the central and significant component of the model before we can arrive at such a unified theory.

Nearly all UK undergraduate students are engaged in employment during their studies and consideration of successful employability post graduation is now a key motivator for undergraduates (Blackwell et al., 2001; Bridgstock, 2009). In light of this, by understanding the needs and expectations of the undergraduate student around employability it is possible to develop a more ecologically sound approach to effective student engagement^1^. The importance of successful entry into the graduate job market is a significant extrinsic motivator for undergraduate students (Cassidy and Wright, 2008). Within the UK HE sector alone there has been an almost universal drive to increase employability skills training and nearly all of the major national key performance indicators include a metric on the vibrancy of a graduating cohorts employability health (Wright et al., 2010). Institutes are readily developing a full range of training opportunities that allow students to develop the authentic and transferable skills that would benefit their entry to the job market e.g., from CV writing advice sessions to entire years of an academic programme spent in structured work placements within industry (Fallows and Steven, 2000; Cassidy, 2006). However the constant aspect that is evident across such diverse innovation is the fact that all place the individual student firmly at the center of the learning experience with idiosyncratic and, in some cases, a highly specific, learning experience that is shaped by the students themselves (Lantz and Reddy, 2010). It is the student that in most cases identifies the type of training hat they need or the location of their placement year. By placing the student at the center of this learning process the design of active learning programmes can benefit greatly from a unique insight into the development of a transferable skill-set (Bridges, 1993). Moreover, the importance of such transferable skills would mean that the self directed nature of such study would be more effective than a more traditional didactic delivery (Gureckis and Markant, 2012). In other words by allowing students the opportunity to reflect on their own employment experiences with other students they are more motivated to understand further the competencies that are effective in work. In light of the above it would be prudent to expand the Rees model to include the unique experience that the student has in the world of work and to design programmes around such experiences.

When one considers the important transferable skillset that the student develops while experiencing an industry placement etc programme managers should place such a student centered experience at the very heart of the learning process (Reddy and Rochelle, 2008). This could take the form of actual work experience or even the design of assessments that are structured around the application of psychological theory to real life work scenarios. This vital aspect of programme engineering can return great dividends with ensuring students are readily recruited to our programmes and can be achieved with surprisingly little effort in a number of stages that ensures the incoming student is readily engaged within an effective learning role (Van De Ven, 2007).

Does a focus on employability favor vocational degrees? Does this threaten Psychology as a non-vocational degree, and the university tradition which is essentially, in the Newman tradition, non-vocational? A response is to argue for “psychological literacy” (see McGovern et al., 2009; Cranney and Dunn, 2011). In essence it suggests that knowledge of research and theory in psychology, and the full methodological range advocated by Rees would strengthen this, allows students to detect false argument better, and become better citizens and employees. An emphasis on employability and psychological literacy is not necessarily in conflict with traditional values; some influential definitions of employability emphasize such scholarly attributes:

“Employability is not just about getting a job. Conversely, just because a student is on a vocational course does not mean that somehow employability is automatic. Employability is more than about developing attributes, techniques or experience just to enable a student to get a job, or to progress within a current career. It is about learning and the emphasis is less on “employ” and more on “ability.” In essence, the emphasis is on developing critical, reflective abilities, with a view to empowering and enhancing the learner.” (Harvey, 2003, cited in Pegg et al., 2012 p. 4 [our bold]).

A further dimension can be added. Barnett (2009) suggests that Higher Education has moved almost by stealth from a focus on knowledge to a focus on skills and competencies. He argues that as we move into a world of further and further complexity neither knowledge nor skills is a secure foundation. He suggests that we need in addition to focus on ontology, on our students' being and becoming. He argues that encounters with disciplinary knowledge through teaching and curricula, such as that in Psychology, support the development of epistemic virtues. A demanding curriculum helps resilience to form; contrasting perspectives help to promote openness; requiring attendance and engagement develops self-discipline; space encourages authenticity and integrity. Teaching that requires students to engage with each other helps to foster respect, generosity and preparedness to listen; explicit standards support carefulness and restraint; encouragement helps to keep students going forward and to be open to new experience; enthusiasm gives new spirit and encourages the will to learn; being required to put forward ones own views helps students to find the courage to stake a claim and to own a position; requiring students to give of selves and be active helps to develop the will to engage.

So—attracting really good students is about the fascination of a subject concerned with what people, think feel and do (not to mention dream, strive for, love and fear). Educating means getting people to be excellent scholars, and translating this into the skills and competencies that employers seek in graduate recruits, helping students to see that psychological literacy and an appreciation of the dialectic in psychology and our methodological pluralism offers valuable ways of seeing and understanding, and helping students to learn from and understand the central importance of being and becoming through study.

By designing academic programmes around the various stages of the learning process described above one can be sure that such programmes are not only effective in delivering a relevant curriculum but one that attracts, engages and retains enthusiastic and talented students to the field. There is no doubt that Rees (2013) should be applauded for initiating the crucial debate as to which factors drive student engagement. Here it is argued that employability training that embeds the student work experience within industry should also be included into this portfolio of effective approaches. The redesign of programmes to includes such strategies may seem complex yet this is the only way to ensure that students are constantly attracted to the degree programmes delivered within universities and then perhaps more importantly remain engaged.

## Footnotes

^1^It is also worth highlighting the utility of this approach in regards to the various national key performance indicators such the National Student Survey in the UK.
